# Local vs. Avatar Robot: Performance and Perceived Workload of Service Encounters in Public Space

**DOI:** 10.3389/frobt.2021.778753

**Published:** 2021-12-03

**Authors:** Jun Baba, Sichao Song, Junya Nakanishi, Yuichiro Yoshikawa, Hiroshi Ishiguro

**Affiliations:** ^1^ AI Lab, CyberAgent, Inc., Tokyo, Japan; ^2^ Graduate School of Engineering Science, Osaka University, Osaka, Japan

**Keywords:** avatar, teleoperated robot, field study, service encounters, performance, workload, customer service

## Abstract

In recent years, the demand for remote services has increased with concerns regarding the spread of infectious diseases and employees’ quality of life. Many attempts have been made to enable store staff to provide various services remotely *via* avatars displayed to on-site customers. However, the workload required on the part of service staff by the emerging new work style of operating avatar robots remains a concern. No study has compared the performance and perceived workload of the same staff working locally versus remotely *via* an avatar. In this study, we conducted an experiment to identify differences between the performance of in-person services and remote work through an avatar robot in an actual public space. The results showed that there were significant differences in the partial performance between working *via* an avatar and working locally, and we could not find significant difference in the overall performance. On the other hand, the perceived workload was significantly lower when the avatar robot was used. We also found that customers reacted differently to the robots and to the in-person participants. In addition, the workload perceived by operators in the robotic task was correlated with their personality and experience. To the best of our knowledge, this study is the first investigation of both performance and workload in remote customer service through robotic avatars, and it has important implications for the implementation of avatar robots in service settings.

## 1 Introduction

In recent years, remote customer service operations *via* teleoperated avatar robots have attracted attention. Avatar robots allow remote service providers to operate robots installed in stores *via* the Internet to interact with customers who visit the stores. Since service providers can respond to customers from their homes or offices as long as there is an Internet connection, this approach is expected to be used as a method of remote work. Avatar robots have been researched for a variety of services, including guidance (Koizumi et al., 2006; Kanda et al., 2010; Heikkilä et al., 2019; Baba et al., 2020), navigation (Glas et al., 2012) in shopping malls, tourist information (Glas et al., 2013), product sales (Song et al., 2021), café clerks (Takeuchi et al., 2020), and expert maintenance (Kritzler et al., 2016).

Remote work has many benefits for both employees and companies, including a better work-life balance for employees (Ferreira et al., 2021), increased productivity (Bloom et al., 2014; Hill et al., 2006; Ferreira et al., 2021), higher employee retention, greater commitment to employing organizations (Golden, 2006; Golden and Eddleston, 2020), a wider talent pool (Kurkland and Bailey, 1999), and prevention of the spread of infectious diseases (Ferreira et al., 2021). In particular, location-based businesses such as retail, which may be restricted by government regulations and authorities from communicating with customers in their facilities to prevent the spread of COVID-19, have recently been exploring methods and practices of remote work (Kim, 2020).

Customer service using avatar robots has been reported to not only allow employees to work remotely but also to be able to perform adequate service tasks. Kanda et al., 2010 confirmed that teleoperated robotic advertising can influence customer shopping behavior, and Song et al., 2021 provided a case of a product sold solely by an avatar robot. Baba et al., 2020 reported that services provided by teleoperated robots can achieve a high level of customer satisfaction. Takeuchi et al., 2020 presented a case study of disabled people working through an avatar robot, which had a positive impact on their mental health; the work performance was also associated with higher frequencies of laughter. While avatar robots have positive effects on both customers and operators, there are reports that the mental workload of operators can lead to poor performance (Landi et al., 2018). Rea et al. (2020), Rakita et al. (2018), and Han et al. (2021) proposed teleoperation interfaces to reduce mental stress. However, all of these studies are aimed at remotely controlling physical robot motions such as carrying and grasping, and have not investigated the workload of robot teleoperation in dialogue-based tasks, such as service encounters, which are subject to interpersonal stress. Providing service tasks *via* avatar robots have the potential to reduce the interpersonal stress of workers because it can create a situation where customers are not physically in front of the workers.

The relationship between the performance and perceived workload using avatar robots is an important indicator for their use in real-world applications. Although robotic avatars have been shown by prior works to perform service tasks well remotely, it remains to be established as to whether this new working style imposes an excessively heavy workload on employees; if so, the practice might negate the evident advantages of remote work, such as improved employee work-life balance and increased productivity. In contrast, if the perceived workload can be reduced with sufficient performance by robotic avatar systems, it could be a factor in promoting the introduction of a new customer service style using avatar robots in businesses. To evaluate the performance and workload associated with the operation of avatar robots, comparing this novel emerging work style with conventional forms of human labor is necessary.

Based on the above, this study addresses the research question regarding the relationship between performance and perceived workload in service encounters in a novel work style with avatar robots compared to the conventional work style. We then form the following hypotheses to address this question.

• H1: There is no difference in the participant performance when providing services through an avatar robot compared to working locally in person.
• H2: Participants’ perceived workload is reduced when interacting through an avatar robot compared to when working locally in person.

We conducted a customer service experiment using an avatar robot in a real public space to verify these hypotheses. In the experiment, we investigated the performance and perceived workload both when the participant provided the service through the avatar and when the same participant provided the service locally without using the avatar. We analyze the results with customers’ reactions and participants’ personalities and discuss the relationship between performance and workload, the differences in customer reactions that contribute to the observed differences in the performance, and the characteristics of participants that relate to the work with the avatar. The key contribution of this study is that it is the first to investigate both performance and workload in providing services *via* avatars, and that it clarifies the advantages and disadvantages of remote employment *via* avatars compared to the conventional work style based on the results of an actual field experiment.

## 2 System

**FIGURE 1:**
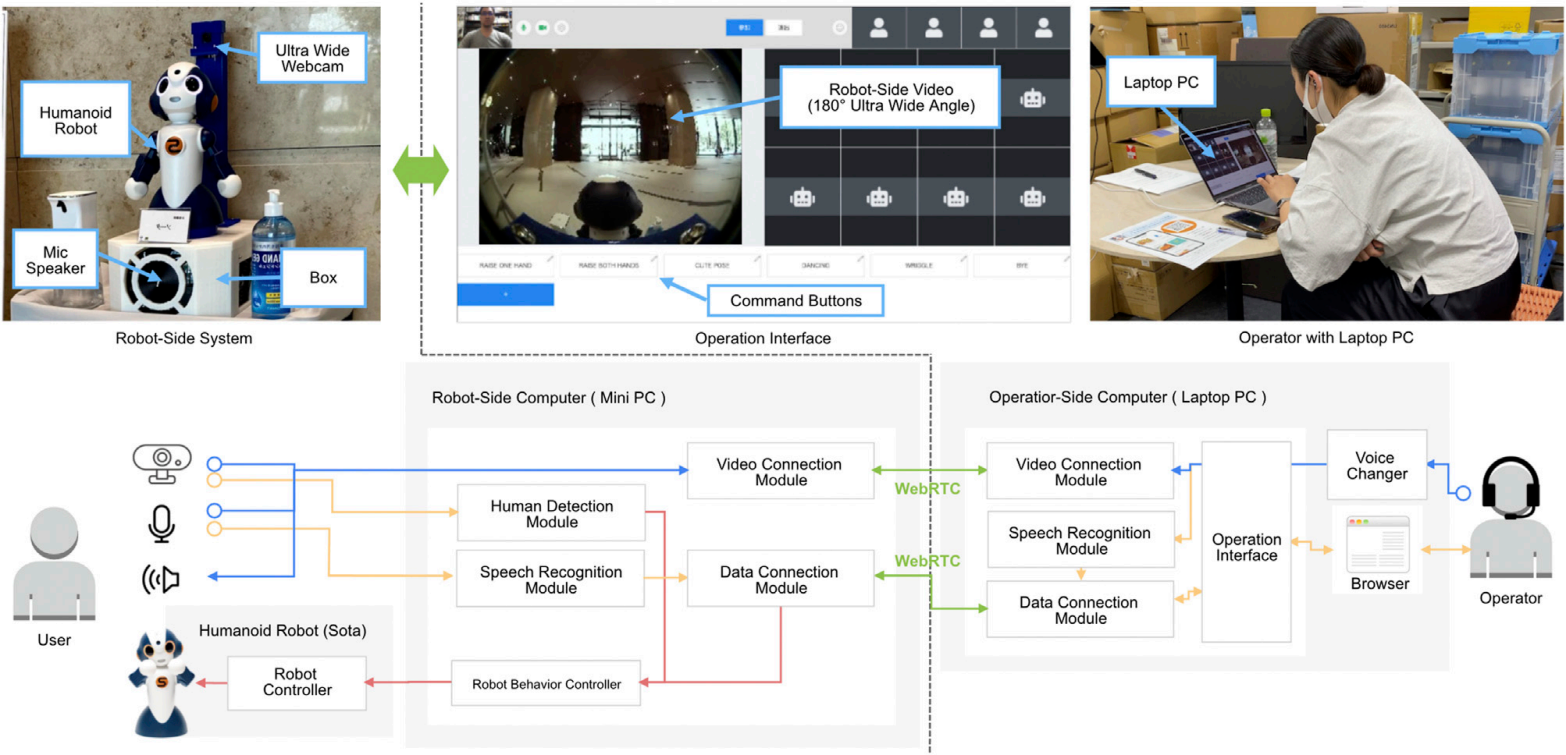
Overview of teleoperated avatar robot system. The operator’s voice commands and clicks are conveyed to the computer on the robot side *via* WebRTC using the data connection module. The voice changer converts the operator’s voice into a robot-like voice, and the converted voice is transmitted to the robot side by a video connection module *via* WebRTC. The transmitted voice is output from the speaker on the robot side, and the robot behavior controller processes the robot’s behavior from the transmitted operation commands.

An overview of our teleoperation robot system is shown in Figure 1. The robot side system consists of a small humanoid robot, Sota (Vstone Co., Ltd.), and a box. The box contains a miniaturized PC to control the robot and video communication, a mic-speaker at the front of the box, and a 180° ultra-wide-angle webcam at the rear. The PC and the robot are connected wirelessly, and the speakerphone and webcam are connected to the PC *via* a USB cable. The program on the PC uses audio and video from the speakerphone and webcam for video communication with the operator side, and sends the commands transmitted from the operator side to the robot. The program running in the robot controls a total of eight motors and blinking LEDs in each eye according to the commands received.

The operator-side system is a web service; the operator accesses the server using a PC browser and opens the operation interface on the browser. The operation interface consists of 1) an operator video area, 2) a robot-side video area, and 3) a command button area. In the operator video area, the video from the operator PC’s input camera is displayed. The operator’s speech is converted to text, and the texts are also displayed in the operator video area to help the operator understand how the system is recognizing their speech. The video on the left side of the display shows the feed obtained from the web camera of the remote robot. When clicking on the video, the click point is displayed in blue, and the corresponding 3D point in the remote robot space is approximately calculated from the two-dimensional coordinates of the point, and the robot turns its face and body in that direction. Finally, each of the command buttons can be used to register a direct command to the robot. When the button is pressed, the robot executes the corresponding movement.

The operator operates the robot in three key aspects. The first is through speech projected by the robot. The content of the operator’s speech is converted into text by Google Speech-to-Text API. If the text contains pre-registered words, the robot performs the action associated with that word. For example, the robot can register pairs of words and actions, such as raising a hand for “hello,” and raising both hands for “welcome” and “thank you.” In this experiment, we registered approximately 40 pairs of words and actions. If there are no words registered in advance, the system performs small, random arm and neck swings in the rest position to express speech gestures when the voice recognition results are returned. In this manner, the robot performs gestures that the operator might perform if they were present in the remote space. Second, the operator can click on the image on the robot side to direct the face and body of the remote robot in that direction. This allows the operator to explicitly control the robot’s gaze, to make face-to-face contact with the user, and to look at the object of their speech. Finally, the operator can explicitly perform a special action by pressing a command button. For example, the operator can perform a special pose or gesture, such as striking a photogenic pose if a user produces a camera to capture a photograph of the robot. We prepared 10 commands for the experiment, such as “raising one hand,” “cute pose,” and “bye.”

## 3 Field Experiment

### 3.1 Task and Design

We conducted the experiment over a period of 10 days from the end of September to mid-October 2020. The experiment was conducted in a new office building that opened in Tokyo in September 2020. The building houses roughly 20 restaurants on the second floor. Many office workers visit those restaurants during their lunch hour. The building is equipped with a system in which the availability of all the restaurants can be viewed in real time on a website.

The participants in this experiment were asked to be avatar robot operators and perform outbound customer service to proactively introduce customers to a QR code to access the website with their smartphones. In this task, the participants spoke to the customers visiting the building to address them and then explain the website and encourage them to access the QR code on the spot. We chose this outbound service owing to its simplicity and uniformity compared to inbound work (Rod and Ashill, 2013), which requires extensive knowledge of a wide range of customer inquiries. Because participants can complete the task with the support of standardized scripts and knowledge of only the service, the work performance is not highly dependent on the expertise and memory of the participants.

**FIGURE 2:**
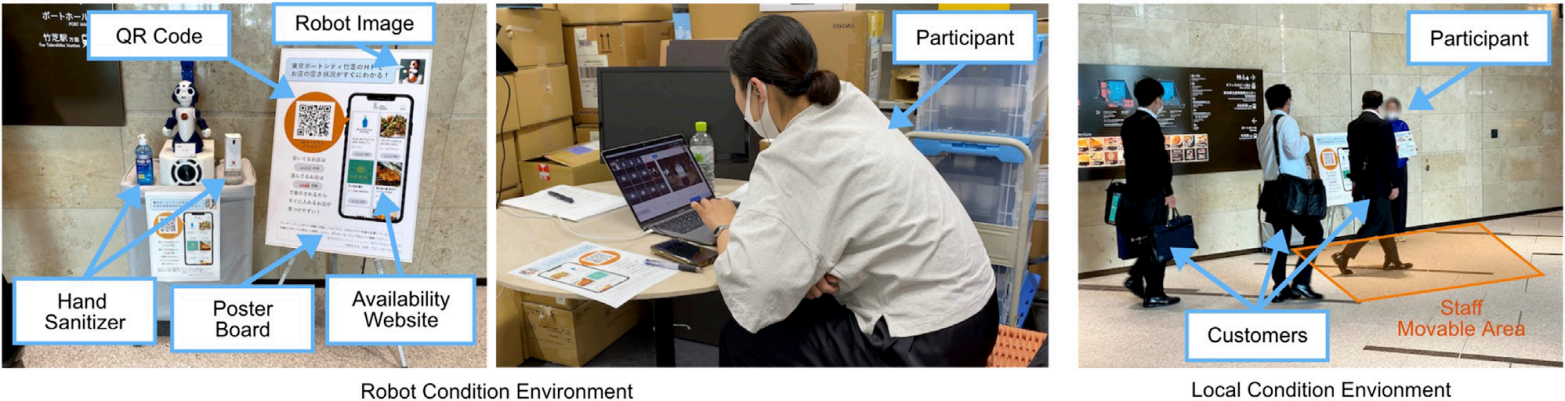
Experiment environments.

The robot system was set up in the environment, as shown in Figure 2. A poster advertising the availability of the website with an image of the robot and the QR code was displayed next to the robot system near the entrance. A hand sanitizer dispenser was placed next to the robot to prevent infection. We designed a within-subject experiment involving both robotic and in-person local working conditions. In the robotic working condition, participants introduced the availability system to the customers through the robot using a laptop PC from a room on a different floor. In the local condition, the same participants themselves stood next to the poster and addressed the customers in person. For this case, the humanoid robot was removed, but the system was left in place to record video. Each working condition was used during 1 h each from 11:30–12:30 and 12:30–13:30 to communicate with customers who came for lunch. The order of the conditions was counterbalanced.

### 3.2 Participants

The participants were recruited through a temporary employment agency to work as part-time participants in the experiment. Ten participants were recruited, five male and five female, eight people were in their 20s, and two were in their 30s. To encourage the participants to actively talk to customers, we designed an additional reward of 100 yen per QR code access, in addition to the wage of 1,100 yen per hour for the participants.

The participants were first given an overview of the experiment and asked to fill out a pre-questionnaire about their experience in customer service and their personality. After responding to the questionnaire, they were presented with the following basic script common to both conditions to ask customers to access the code.

1. Communicate what you are introducing in an easy-to-understand manner. (e.g., “This is a website that shows restaurant availabilities!”)
2. Promote the features and benefits. (e.g., “You can see instantly which restaurants have seats available now!”)
3. Give customers permission to take photographs of you/the robot to encourage them to take out their smartphones. (e.g., “You can take a picture of this robot/poster!”)
4. Request specific actions from customers. (e.g., “Take out your phone and read this QR code right here!”)

We allowed the participants to improvise words not in the script and chat with customers freely. We also told them that even if there was no one nearby, constant talking could bring in customers from far away. In both conditions, the participants were allowed to ask customers to disinfect their hands with the sanitizer near the robot as a chance to talk. In the local condition, the participants were given an A4-size pop-up so that people could easily identify them as staff, and were also instructed that they could move within an area of approximately 3.0 m × 1.5 m around the side of the poster shown in Figure 2 in order to align the environment for both conditions. In the robotic working condition, participants were told the robot’s assigned name, age, and hometown, and were instructed to act naturally while performing as the designated robot character when operating it to avoid negative reactions from customers. We told the participants that as long as they were consistent with the robot’s character information, they would have no problem speaking fluently and talking about themselves in their normal manner.

We announced to all pedestrians through a notification board that this was an experiment, and that video was being recorded by the web camera on the robot system. This study was conducted on an opt-out basis for unwilling participants who chose to be removed from the video data. This experiment was approved by the facility authorities in the office building and the Research Ethics Committee of Osaka University (Reference number: R-1-5-4).

### 3.3 Measurements

The pre-experiment questionnaire given to the participants contained questions regarding their years of customer service experience and personality. We used the Japanese version of the Ten-Item Personality Inventory (Oshio et al., 2012) to measure the Big Five personality dimensions (Goldberg, 1990), including extraversion, agreeableness, openness to experience, conscientiousness, and neuroticism.

To compare the two conditions, we measured task performance, perceived workload, and customer response. For task performance, we used three indices: the stop rate, access rate, and whole access rate. The stop rate is the ratio of the number of people that stopped to the number of all passersby. The access rate is the ratio of the number of people who produced their smartphone and accessed the QR code to the passersby that stopped. The whole access rate is the ratio of the number of people who accessed the QR code to the total passersby. In the experiment, we placed a counting person in a position where they could observe the entire environment, and the same person directed the experiment. They counted the number of passersby, the number of people who stopped in front of the robot/participant, and the number of people who accessed the QR code. To clarify the count targets, they counted as passersby those who passed through an area of approximately 7 m square enclosed by large pillars on either side in front of the robot and the wall behind the robot. Customers who stopped in front of the robot while looking at the robot or participant were counted as “stopped.” Access to a QR code was counted when both the customer pointed their smartphone’s camera at the robot/participant and the notification was pushed when the QR code was accessed.

The Japanese version of the NASA-TLX was used to measure participants’ perceived workloads (Haga and Mizukami, 1996). The NASA-TLX has six categories of demands: mental demand, physical demand, temporal demand, overall performance, effort, and frustration level. The participants were first asked to rate which category they felt was more important. Specifically, since there are 15 possible combinations of the six categories, the participants were asked to make 15 comparative judgments. We counted how many of each category they judged to be more important and normalized the value to between 0 and 1 as the weight for each category. They were then asked to score the degree to which they felt the six demands were required on a linear scale. This was then calculated as a score on a 100-point scale. After the participants had scored all the items, they were asked to provide a written reason for the score they assigned to each item. Using the normalized weights for importance perceived by the participants, the average of the weighted scores was used as the overall workload.

To record customer reactions, we randomly asked customers who stopped in front of the robot/participant to fill out a questionnaire as shown in Table 1. Q1 was used to check whether the customer correctly recognized the QR code introduced by the participant. Q2 and Q3 were used to check the reason for the customer’s behavior.

**TABLE 1:** The content of customer questionnaire.

No.	Question	Answer type
Q1	What was the QR Code that was being introduced about?	Free text
Q2	Did you access the QR code?	Yes/No
Q3	Why did you access or not access the QR code?	Free text

To analyze the customer’s answers written in free text, we tasked two coders to annotate the answers. One coder was the author, J. B., who created the codebook before annotating. The other was hired as a part-time worker, and annotated the answers according to the codebook. The codes for Q1 consisted of “Correct,” “Incorrect,” “I don’t know,” and “Unjudgeable.” The codes for Q3 consisted of “Mention a recommender,” “Mention a feature/benefit of the site,” “Mention a customer’s situation” and “Unjudgeable.” For example, “Mention a recommender” was given as a reason related to the interaction between the customer and the recommender or the impression of the robot/staff, such as “because the staff recommended it to me” or “because the robot was cute.” The “Mention a feature/benefit of the site” code was assigned to record reasons related to the functions and features of the website, such as “because the site is convenient,” or “to check which restaurants have seats available.” The “Mention a customer’s situation” code was assigned for reasons related to the situation of the customer, such as “because I am in a hurry,” or “I have already had lunch.” Both coders annotated all data, and the overlapped data showed that they substantially matched (Cohen’s Kappas for Q1 and Q3 were 0.768 and 0.854, respectively), and the data coded by the hired coder was used in the analysis.

## 4 Results

### 4.1 Task Performance

**FIGURE 3:**
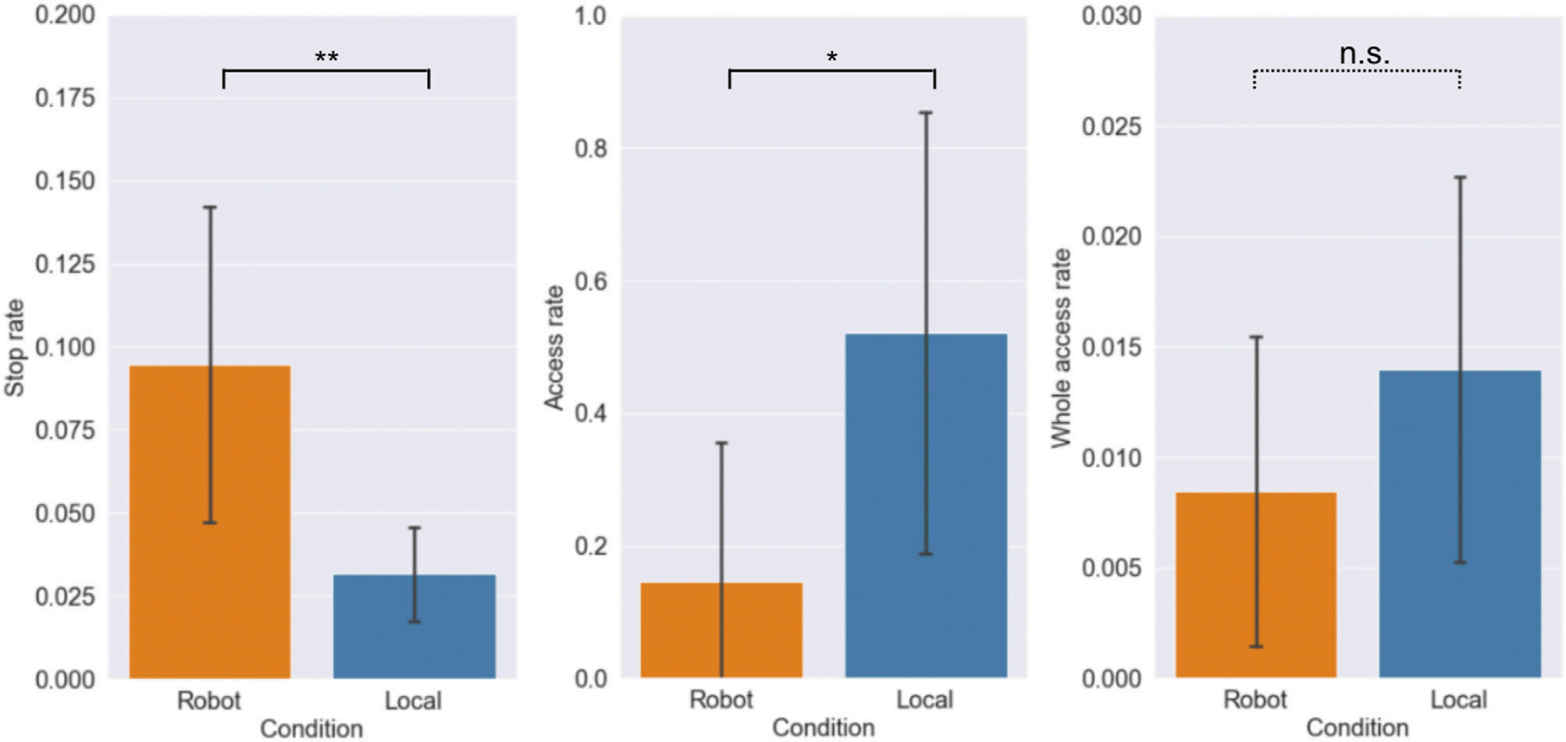
Averages of participant’s task performance. Error bars represent SD. ** and * indicate significant differences in *p* < .01 and *p* < .05 between the robot and local conditions, and *n*.*s*. indicates that there was no significant difference between the two conditions. Note that the scales of the axes are different due to the different denominators of each rate.

Figure 3 shows the results of the average task performance under both conditions. The average stop rates for the robot and local conditions were 9.47 and 3.16%, respectively. There was a significant difference between the two conditions (Wilcoxon’s signed rank sum test, *T* = 1.0, *p* = 0.004 < 0.01). The average access rates for the robot and local conditions were 14.66 and 52.08%, respectively. There was a significant difference between the two conditions (Wilcoxon’s signed rank sum test, *T* = 7.0, *p* = 0.037 < 0.05). The average whole access rates for the robot and local conditions were 0.84 and 1.40%, respectively. There was no significant difference between the two conditions (Wilcoxon’s signed rank sum test, *T* = 14.0, *p* = 0.193). Based on the above results, the participants in the robot condition stopped more customers than in the local condition; however, fewer stopped customers accessed the QR code than in the local condition. Although the overall performance, whole access rate, in the local condition was slightly higher than that in the robot condition, we could not confirm a significant difference between the two.

### 4.2 Perceived Workload

**FIGURE 4:**
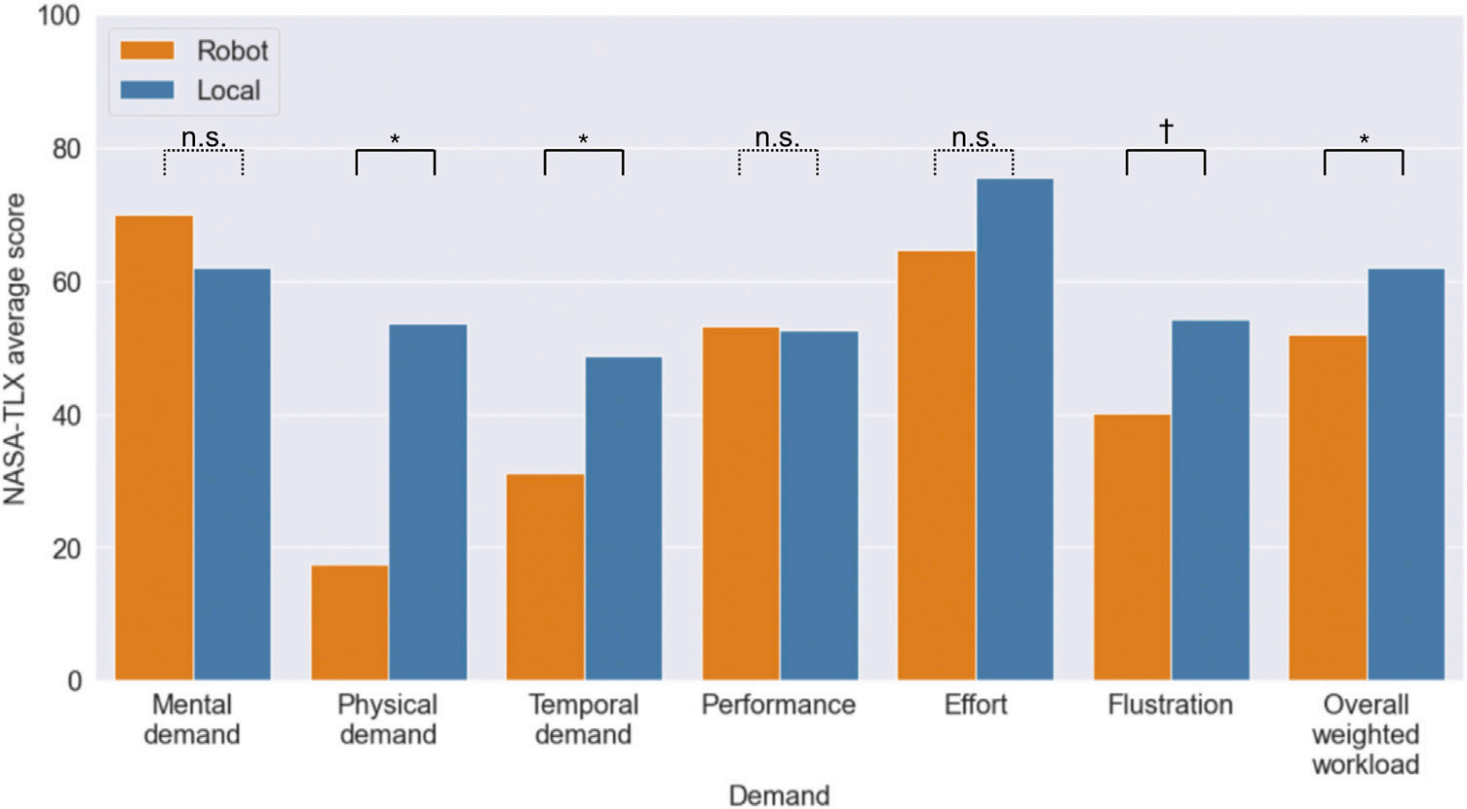
Average scores of NASA-TLX. * and † represent a significant difference and a significant trend in *p* < .05 and *p* < .10 between the robot and local conditions, and *n*.*s*. indicates that there was no significant difference between the two conditions.

The results of the perceived workload for each demand and the overall weighted workload are shown in Figure 4. The mean mental demand scores for the robot and local conditions were 70.0 and 61.90, the mean physical demand scores were 17.5 and 53.71, the mean temporal demand scores were 31.12 and 48.62, the mean performance scores were 53.28 and 52.59, the mean effort scores were 64.74 and 75.52, the frustration score means were 40.17 and 54.22, and the overall weighted workloads were 53.33 and 60.55, respectively. The robot condition was less demanding than the local condition for all items except mental and performance demands. Analyzing the results of the Wilcoxon’s signed rank sum test, there were significant differences in physical demand (*T* = 2.0, *p* = 0.015 < 0.05), temporal demand (*T* = 5.0, *p* = 0.038 < 0.05), and overall weighted workload (*T* = 5.0, *p* = 0.02 < 0.05). There was also a significant trend in frustration scores (*T* = 8.0, *p* = 0.0858 < 0.10).

We computed the Spearman’s rank correlations between the perceived workload and the attributes of the participants in both conditions. As indicated by the results shown in Table 2, the extraversion and years of customer service experience were significantly and negatively correlated with the OWW only in the robot condition, and there were no attributes with significant correlation coefficients with the OWW in the local condition. The years of customer service experience were also significantly and negatively correlated with the frustration level only in the robot condition. There were significant correlation coefficients between agreeableness and frustration, and between neuroticism and effort demands.

**TABLE 2:** Spearman’s rank correlation coefficients between the participant’s attributes and the perceived workload demands. Attributes are indicated by the initial letter: (Y) = years of customer service experience, (E) = extraversion, (A) = agreeableness, (O) = openness to experience, (C) = conscientiousness, and (N) = neuroticism. The columns are the NASA-TLX demands, and OWW is an abbreviation for the overall weighted workload. * indicates a significant correlation in *p* < .05.

Robot condition									
Attribute		Mental	Physical		Temporal	Performance	Effort	Flustration	OWW
(Y)		0.2263	−0.3034		0.1835	−0.5644	−0.0917	−0.7523*	−0.7646*
(E)		−0.1371	−0.0442		0.4425	−0.1876	−0.4301	−0.3864	−0.6981*
(A)		−0.6050	−0.0750		−0.0062	0.3685	0.4877	−0.2531	0.0062
(O)		−0.0432	−0.1188		0.0432	0.2384	0.0741	−0.4075	−0.1605
(C)		−0.2920	−0.2453		0.1056	0.2867	0.0062	0.0683	−0.1678
(N)		0.2805	−0.0247		−0.1220	0.0061	0.1402	0.0976	0.6037

Local condition									
Attribute		Mental	Physical		Temporal	Performance	Effort	Flustration	OWW
(Y)		0.0123	−0.2699		−0.4282	−0.2447	−0.3466	−0.3731	−0.4404
(E)		0.1250	−0.2470		−0.5859	−0.1122	−0.4001	−0.2618	−0.4550
(A)		0.2601	−0.5666		−0.5865	0.2902	−0.0836	−0.4322	−0.2284
(O)		0.3468	−0.1765		−0.5248	0.3025	0.1579	−0.3210	0.0000
(C)		0.3988	0.0748		−0.5157	0.1056	0.1246	−0.1678	−0.0932
(N)		−0.0336	0.4312		0.4756	0.0244	0.7187*	0.3720	0.4939

### 4.3 Customer Reactions

**FIGURE 5:**
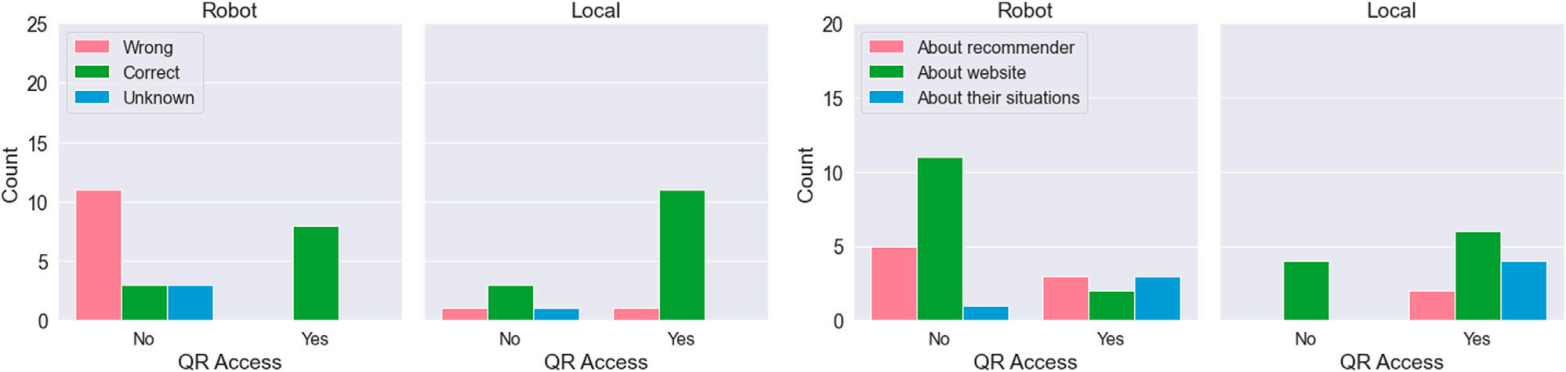
Customer responses to “Q1: What was the QR Code that was being introduced about?” and “Q3: Why did you access or not access the QR code?” Statistical tests were not conducted because of the small number of responses by factors.

A total of 158 customers were approached, and 49 (31%) responded to our questionnaire. The customers who responded included 22 males and 27 females, and their ages varied as one teenager, eight people in their 20s, 11 people in their 30s, 12 people in their 40s, 11 people in their 50s, and 5 people in their 60s. Figure 5 shows the results tabulated for each condition and QR access, excluding “Unjudgeable” from the codes for each question. As shown on the left in Figure 5, most of the customers in the local condition correctly understood the contents of the QR code, while those who did not access the QR code in the robot condition hardly understood the QR code. The figure on the right also shows that the percentage of “Mention a recommender” increased in the robot condition compared to the local condition.

## 5 Discussion

There were significant differences in the partial performance between the two conditions. The stop rate was higher in the robot condition, whereas the access rate of the customers who stopped was higher in the local condition. In other words, the avatar robot could stop more customers and take more opportunity to communicate with them, but the local staff could encourage more stopped customers to take the desired action than the avatar robot. Although there were differences in each condition, they were offset and as a result, no significant differences in the whole access rate, the overall performance, were confirmed in this experiment. For other tasks, there will be some variation in the performance difference depending on how they are offset. Still, the offsetting will not make a huge difference in performance. The results of the study by Tonkin et al., 2017 also showed that the maximum performance in both robot and staff conditions was the same. At least for the task of outbound customer service, as in this experiment and Tonkin’s study, we could not find any significant difference in performance between working remotely with a robot and working locally, and H1 was not denied.

These different features in partial performance can be attributed to the higher ability of humanoid robots to attract people’s attention. It has been reported that robots can attract the attention of users by moving and talking, and robots can be used to stop many people (Okafuji et al., 2021). However, this ability includes the problem of attracting too much interest. In this experiment, it was found that the access rate in the robot condition was lower than that in the local condition, but also that most customers did not understand the content of the QR code introduced by the robot. It was also found that a larger percentage of customers mentioned the recommender as the reason for their behavior in the robot condition. For example, customers gave the following reasons for accessing the QR code: “The robot was cute and stopped me in my tracks,” and “Sota recommended it to me.” They also gave answers for not accessing the code such as, “The robot suddenly started doing business.” This result suggests that the customers’ attention was focused on the robot and not on the content that the robot introduced. Similar results were confirmed in the studies of Song et al. (2021) and Tonkin et al. (2017), and this experiment also reaffirmed the major challenges of proactive service delivery by robots.

In this experiment, we investigated the perceived workload as well as the performance. The results show that the overall weighted workload was significantly lower in the robot condition than in the local condition, and H2 was supported. From these results, we can state that avatar robots in service encounters have the advantage of reducing perceived workload while having the potential to maintain performance. This is the main contribution of this study, and is a very important finding for companies and researchers aiming to implement avatar robots in real-world applications.

The lower workload in the robot condition was due to the fact that the physical and temporal demands were significantly lower. One possible reason for the lower physical demands was that the participants did not stand and move their bodies, but only sat in chairs and talked. In the questionnaire, the participants who rated the physical demands as low said, “I didn’t have to move my body because I just sat and talked,” and “It wasn’t painful to sit in the chair.” Because service work involves a considerable amount of standing and moving physically, it can be said that service work through an avatar robot, the main physical activity involved in which is only to sit in a chair and talk remotely, requires less physical labor than the conventional working style. This is not limited to avatar robots, but is a characteristic of remote customer service in stores using video conferencing tools. Next, there may be two factors for the low temporal demands. First, participants were not physically close to the customers, and they were thus less likely to feel direct pressure from the customers. A participant commented, “I didn’t feel much time pressure because rushing won’t change anything,” suggesting that they did not feel the need to rush to meet customers. As another factor, it could be said that customers interacting with the avatar robot were not in a hurry and had sufficient time to talk with the robot. As mentioned above, in the robot condition, the customers paid attention to the robots, and as far as the experimenter observed, many customers seemed to enjoy chatting with the robot. The customers may not have felt much pressure from the robot, as they did not really comply with the robot’s request for QR code access, despite stopping long enough to have a conversation. This could be a feature of the new working style involving avatar robots. In addition, the perceived mental demand in the robot condition was slightly higher than that in the local condition. This result can be caused by two operations: carefully watching the robot video to grasp the customer’s facial expressions and considering speech to attract and persuade customers. The participants also commented, “Because I looked carefully at the information from the images and thought carefully about how to talk about stopping them,” and “It was difficult to read the information from the screen and to think about the words to be spoken because I was not face-to-face with customers.” This slight increase in mental demand can be seen as a new added workload that should be a concern in the new work style with avatar robots. Although this point needs to be carefully examined, it did not have a large impact on the overall workload, it was found that the benefits in terms of physical and time pressure had a greater impact on the overall workload. It is not clear whether these workload characteristics were due to the avatar robot or video conferencing installed in the system, and further investigation is needed. Nevertheless, it can be considered that remote work through avatar robots has a certain advantage when compared to the conventional style.

In the robot condition, the overall weighted workload was significantly correlated with the attributes of the operator. It was clear that the robot workload was lower when the participants had more years of customer service experience or when the participants were more extroverted. It was also confirmed that those with more years of service experience felt less frustrated when working through the robot. The frustrations felt in this outbound task include 1) tension and anxiety about proactively talking to the passersby and 2) frustration involved in negative reactions from customers (Yagil et al., 2008; Karatepe et al., 2010; Sliter et al., 2011). Experienced participants were more likely to be accustomed to the first frustration, and this telework with an avatar robot may have significantly reduced the second frustration because they felt fewer negative reactions due to the increased social distance of video-based interaction. In fact, some experienced participants commented on the robot condition, “Because the video is passed through the robot, the reactions of the customers do not affect the mind,” and “I didn’t actually have a customer in front of me, so I was comfortable with that.” Furthermore, in the local conditions, they made comments such as, “Because it hurts my feelings a little when people ignore me in person,” and “I feel that directly ignoring me hurt my feelings more than when operating the robot.” In contrast, inexperienced participants responded that “the work was performed under a lot of tension” and “I have a hard time talking to people because I’m not very good at it,” indicating that they were focused on the work or talking. Therefore, the participants with more years of experience may have felt fewer types of frustration, and the majority of the frustration, “feeling negative reactions from customers,” was reduced when working through an avatar robot. The extroverts may have felt less frustration and effort demands because they enjoy interacting with people, and many customers chatted with them in the robot condition. The comments of the highly extroverted participants included “It was interesting to me,” and “I was able to enjoy the work because I talked like a robot,” indicating that they enjoyed conversations with customers.

In the local conditions, the participants with high neuroticism felt that considerable effort was required. This could be due to the fact that many of them were nervous about having face-to-face conversations with customers, and it is possible that they needed to make an effort to perform their duties in this situation. There were also comments from participants with high neurotic tendencies, such as “I felt very high effort demands because the work was done under high tension” and “I felt that effort was quite necessary because my impression and attitude would come out.” However, in the robot condition, the same people commented, “I was less tense because I did not see others face to face” and “The sense of urgency was relaxed and I was able to work at a slower pace.” Although a contrary significant correlation was not found in the robotic working condition, these comments suggest that people who are easily stressed may be able to reduce their stress by working with an avatar robot.

These findings regarding the relationship between the attributes of the participants and the perceived workload are expected to be useful in determining compatibility between the work and the operators required for avatar robots. People with many years of experience in customer service and high extraversion, and people with high neurotic tendencies may be suitable for remote work through avatar robots, and novel systems to reduce the perceived workload to support others are needed.

## 6 Limitaion

There are several limitations of this study. The first major limitation is the small sample size of the participants. For example, the number of participants was 10 in this experiment, and the average number of QR accesses was 1.55, indicating that the sample size may be insufficient because of the small number of accesses. For the items for which no significant difference was found, the confidence level may not be sufficiently high. Similarly, by increasing the sample size to examine the significance of the differences in mental demands and correlation coefficients, we may be able to confirm significant differences that were not revealed in this experiment. This is a main future work and requires further investigation.

We used only small humanoid robots as avatar robots in this experiment. Because it has been reported that people’s impressions of robots differ depending on their appearance, size, embodiment, and emplacement (Tanaka et al., 2014; Li, 2015), it could partially change the claims of our study. However, the results on workload that we were able to confirm in this experiment were largely influenced by the non-face-to-face nature of the video call, and we consider that the appearance of the robot had little effect.

In addition, the task examined was an outbound service in which the customer was approached and made aware of a service, and inbound tasks were not considered. More to the point, It is not clear that the findings of this study could generalize to complex teleoperation tasks, such as making the robot perform the physical work. Furthermore, this experiment was conducted in Japan; the participants were only in the younger age group, and the customers were mainly office workers around the building. Hence, the attributes of the participants and customers were biased to some extent, and we cannot explain the extent to which age, occupation, and cultural differences may have affected the results.

## 7 Conclusion

In this study, we conducted a field study to compare task performance and perceived workload, which are important indicators in the implementation of avatar robots in real-world applications, in which participants provided services remotely *via* an avatar robot and also worked locally in person. There were significant differences in the partial performance between the robot condition and the local condition, and we could not find significant difference in the overall performance. On the other hand, there was a significant difference in the overall workload, confirming that the workload was lower when using an avatar robot. In addition, customers responded more strongly to the avatar robot than to the in-person staff. However, they were too focused on the robot to be aware of the service content. With regard to the perceived workload, we found that mainly physical and time demands decreased in the robot condition, and that there were significant correlations between the workload and the attributes of the operators. The advantages and disadvantages of avatar robots revealed in this study not only provide important insights for the business use of avatar robots, but also provide a starting point for investigating the effects of avatar robots on users and operators.

## Data Availability

The original contributions presented in the study are included in the article/supplementary material, further inquiries can be directed to the corresponding author.
